# Assessing the Impact of a School Intervention to Promote Students’ Knowledge and Practices on Correct Antibiotic Use

**DOI:** 10.3390/ijerph10072920

**Published:** 2013-07-15

**Authors:** Maria-Manuel Azevedo, Céline Pinheiro, John Yaphe, Fátima Baltazar

**Affiliations:** 1 School E.B. 2,3 D. Maria II, Rua da Alegria, Vila Nova de Famalicão 4760-067, Portugal; E-Mail: maria.manuel.azevedo2011@gmail.com; 2 Department of Microbiology, Faculty of Medicine, University of Porto, Porto 4200-319, Portugal; 3 Life and Health Sciences Research Institute (ICVS), School of Health Sciences, University of Minho, Braga 4710-057, Portugal; E-Mails: celinepinheiro@ecsaude.uminho.pt (C.P.); yonahyaphe@hotmail.com (J.Y.); 4 ICVS/3B’s-PT Government Associate Laboratory, Braga/Guimarães 4710-057, Portugal

**Keywords:** antibiotics, knowledge attitudes/practices on antibiotic use, middle-school students, school teaching intervention

## Abstract

The clinical efficacy of antibiotics depends on their correct use. Widespread ignorance and inappropriate attitudes to antibiotic use have been identified among consumers. In order to improve the knowledge of middle-school students on antibiotics and their correct use, 82 ninth-grade students were enrolled in a teaching activity. The teaching activity consisted of a slide show presentation followed by discussion in a regular class. To evaluate the impact of the teaching activity the students were asked to answer a questionnaire before and after the activity. This study aimed: (1) to evaluate knowledge on the use of antibiotics in students of two schools in the north of Portugal and (2) to evaluate the efficacy of the school intervention in improving students’ knowledge on correct antibiotic use. We found lack of knowledge among students regarding antibiotic spectra and indications and incorrect attitudes in the pre-test. Significant increases in knowledge were observed after implementation of the teaching activity. Knowledge of the correct use of antibiotics for bacterial diseases rather than viral diseases rose from 43% to 76% in the post-test (*p* < 0.01). Knowledge of the risk of bacterial resistance to antibiotics from their incorrect use rose from 48% to 74% in the post-test (*p* < 0.05). We believe that it is important to reinforce the teaching activities on microbiology and antibiotic use at the middle school level.

## 1. Introduction

Antimicrobial resistance is a current problem, including the treatment of multidrug resistant bacterial infections and the prevention of the spread of resistant microorganisms. The World Health Report 2007 highlighted the issue of antibiotic resistance as one of the major threats to public health in this century [1]. The World Health Organization estimates that about half of all medicines are inappropriately prescribed and about half of the patients fail to take their medicines properly.

It is known that the clinical efficacy of antibiotics depends greatly on their correct use. This is dependent on the patients, physicians and retailers [2]. Widespread problems in knowledge, attitudes, beliefs and behaviors have been reported among consumers. These can influence correct antibiotic usage [3,4,5,6]. In the UK, 30% of adults believe that antibiotics can be successfully used to treat coughs and colds [4,7]. A study performed by Eurobarometer in 2001, revealed that 60% of the Europeans do not know that antibiotics are ineffective against viruses [8]. Overuse of antibiotics has been reported in upper respiratory tract infections, despite the fact that the majority of these infections are caused by viruses [9,10]. A recent study confirms that the expectation of antibiotic efficacy for common cold symptoms is very high (47.3%) [11]. This result is comparable with a study conducted in the US, which reported that 48% of the respondents ask for antibiotics for cold symptoms [3].

Physicians’ decisions may be influenced by several factors such as the fear of losing the patient’s trust, a lack of correct information on indications for antibiotic use, and pressure from patients and family [12,13]. Patient expectations influence antibiotic prescription and antibiotics are more likely to be prescribed in a pressured clinical context [13,14,15]. A survey of medical students in the Northeast of the United States indicated that there is a need for both education and feedback on antimicrobial prescribing [16]. A recent study conducted in China reveals widespread prescribing of unnecessary antibiotics, often administered parentally [17]. Other studies show that mothers often influence medical decisions on antibiotic prescription [18].

In this context, health promotion interventions in this particular issue are crucial. Efforts to reduce antibiotic resistance should include educating the population for the appropriate use of antibiotics. Several countries have developed campaigns to modify public misconceptions regarding the effectiveness of antibiotics, to promote appropriate use of antibiotics and prevent the development of antibiotic resistance [19,20,21]. In Malaysia, the “Know Your Medicine Campaign” that aimed to educate and prepare the public with knowledge and skills to understand their medicines, to use medicines rationally and to know their rights about getting information on medicines [22], has recently focused on antibiotics. Belgium has organized several national media campaigns to alert the public to the problem of antimicrobial resistance and has developed guidelines for ambulatory care physicians [23]. Perhaps the largest education effort directed at students is the e-Bug project. This includes 10 European countries, including Portugal, as associates, and has 8 additional countries as collaborators. It includes classroom teaching materials, games and a website designed to increase knowledge of infectious diseases and antibiotic treatment among school age children [24]. In France, the rates of antibiotic prescriptions and antimicrobial resistance are also significant [25]. Since 2000, various local and national interventions have been initiated in order to promote appropriate antibiotic use, such as “Antibios quand il faut” [26] and “Antibiotiques c’est pas automatique” [27]. These interventions appeared to be successful since there has been a decrease of 26.5% in community-based antibiotic prescribing between 2002 and 2007 [27]. France has participated in the European e-Bug school project [28] to raise awareness among children and to help them to adopt correct attitudes and behaviours. The enthusiasm and collaboration between educational and health partners have been obvious [28].

In Portugal, studies performed in schools at different educational levels have also shown a lack of knowledge of antibiotics and their correct use. This may be attributed to insufficient formal education on this topic [29]. In the Portuguese biology curriculum, the concept of antibiotics is only addressed in the 12th grade biology course [30]. This reinforces the importance of developing health education programs to promote appropriate use of antibiotics for middle-school students.

As students are the antibiotic users of tomorrow, it is crucial to invest in their education. The misuse of antibiotics may lead to considerable risks. Effective action requires educational procedures based on the study of existing beliefs.

Three questions motivated the current study. How accurate is the knowledge of middle school students on correct antibiotic use? Are there differences in the knowledge of students in urban and rural schools? What is the contribution of a teaching activity to the students’ awareness and understanding about antibiotic use and resistance? Thus, this study aimed to evaluate: (1) knowledge concerning the use of antibiotics in Portuguese students aged 14 to 16 years old in the ninth grade of two schools (School D. Maria II and Camilo Castelo Branco) from Vila Nova de Famalicão, Braga, Portugal and (2) the efficacy of a teaching intervention in improving students’ knowledge concerning antibiotic use.

## 2. Experimental Section

### 2.1. Population and Sampling

A convenience sample of middle-school students was used in this study. Information about possible confounders, such as socio-economic status and intellectual level, was not collected. The sample comprised 82 students from 9th grade (37 from the School E. B. 2, 3 D. Maria II, located at about 2 km from the city of V. N. Famalicão, district of Braga, and 45 from the School Camilo Castelo Branco located in the centre of the same city). The first school receives students from the center of the city and the second from more rural and manufacturing areas. Approval was obtained from the directors of the schools involved in the study.

### 2.2. Questionnaire

The students’ knowledge was evaluated with a questionnaire validated in a previous study [29], before and after the implementation of the teaching activity. The questionnaire included questions regarding knowledge and attitudes on appropriate use of antibiotics and antibiotic resistance. The time between the implementation of the activity and the post-test application was 2 months. The questionnaire contained seven questions. The first two questions containing five items are related to the link between the use of antibiotics and microorganisms or diseases. The next five questions are related to the correct utilization of antibiotics.

No further changes were made in this instrument. The questionnaire was completed during regular classes with a time limit of 30 min. Participation was voluntary and anonymous. The questionnaire was designed to assess the effect of a teaching activity on student’s knowledge concerning antibiotic utilization. This study instrument also allowed assessment of differences in knowledge between the two schools. Individual papers were not matched for the pre- and post-test to preserve anonymity and scores for the whole class were compared.

### 2.3. Teaching Activity

The teaching activity consisted of a presentation (a computerised slide show), followed by discussion in a regular class lasting 90 min. The first part of the presentation contained basic information on microorganisms such virus, bacteria, fungi and protozoa. The second part was related to diseases and focused on the use of drugs against the different types of microorganisms such as viruses, bacteria and fungi. Particular attention was given to the treatment of diseases caused by bacteria, especially tuberculosis. The presentation centered the attention on antibiotics, including: (1) the story of the discovery of antibiotics, (2) the effectiveness of antibiotics against bacteria, (3) the correct use of antibiotics and, (4) the recent problem of antibiotic resistance. After the presentation, the two teachers (MMA, FB) involved in this study discussed (1) the use of microorganisms in industry, health and research, (2) negative aspects of microorganisms for human health such as diseases and (3) the correct use of antibiotics and the problem of antibiotic resistance. FB teaches Pharmacology at the Medical School at the University of Minho, Braga, Portugal and MMA does research at the Microbiology Department of the Faculty of Medicine in the University of Porto, Portugal and teaches at D. Maria II School, Famalicão, Portugal.

### 2.4. Data Analysis

Data obtained in the pre- and post-test were analysed using the the SPSS software for Windows, version 18.0 (SPSS Inc., Chicago, IL, USA). Associations between variables were tested with Pearson’s Chi-square (χ^2^) with significance set at the *p* < 0.05 level.

## 3. Results

The response rate was 100% among the 82 students asked to participate. 37 students from School D. Maria II and 45 from School Camilo Castelo Branco aged between 14 and 16 years old were enrolled in this study.

### 3.1. Knowledge on Antibiotic Use against Bacteria and Other Organisms

Data presented in Table 1 show the knowledge of students on the sensitivity of different organisms to antibiotics. There were high percentages of incorrect answers among all the students evaluated in the pre-test, with only 2.7 and 0% of correct answers for Schools D. Maria II and Camilo Castelo Branco, respectively. Comparing with the post-test, although not significant, there was an increase in the number of correct answers (Option 1) in both schools, while there was an increase in the answers for Option 2 (incorrect) and a decrease for Option 3 (incorrect). However, the only statistically significant difference was observed for Option 3 in School Camilo Castelo Branco (*p* = 0.010).

**Table 1:** Number of positive answers to the question on antibiotic use against bacteria and other organisms.

Schools	n	Options								
		Option 1: Antibiotics are effective against bacteria only * n (%)			Option 2: Antibiotics are effective against bacteria and other organisms n (%)			Option 3: Antibiotics are ffective against other organisms n (%)		
		*Pre-test*	*Post-test*	*p* value	*Pre-test*	*Post-test*	*p* value	*Pre-test*	*Post-test*	*p* value
D. Maria II	37	1 (2.7)	3 (8.1)	0.615	4 (10.8)	6 (16.2)	0.736	32 (86.5)	28 (75.7)	0.235
Camilo Castelo Branco	45	0 (0)	3 (6.7)	0.242	7 (15.6)	15 (33.3)	0.050	38 (84.4)	27 (60.0)	0.010

* Correct answer.

### 3.2. Knowledge of Antibiotic Use against Viral and Bacterial Diseases

Data presented in Table 2 show the knowledge of students on antibiotic use against viral illnesses (influenza, hepatitis and AIDS) and tuberculosis. In the pre-test, students revealed lack of knowledge in this topic, since only 40.5% and 46.7% of the students respectively from the School D. Maria II and Camilo Castelo Branco answered correctly (Option 1). In the post-test a significant increase in the number of correct answers was observed for both schools (*p* = 0.005 and *p* = 0.001, for D. Maria II and Camilo Castelo Branco, respectively). Regarding the incorrect answers, there was a significant decrease in the choice of Option 2 (*p* = 0.005 and *p* < 0.001, for D. Maria II and Camilo Castelo Branco, respectively).

### 3.3. Attitudes towards Antibiotic Use

Data presented on Table 3 show the knowledge of the students concerning correct antibiotic use. Comparing the pre-test and post-test, significant differences were found in Question 2, for the students of both schools, with an increase from 40.5% to 73% (*p* = 0.005) in the percentage of correct answers for School D. Maria II and from 46.7% to 82.2% for School Camilo Castelo Branco (*p* = 0.002). Regarding Question 5, an increase in correct responses between the pre- and post-test was obtained for students from both Schools but this difference was not significant.

**Table 2:** Number of positive answers to questions on antibiotic use against viral illnessess (influenza, hepatitis, AIDS) and tuberculosis.

Schools	n	Options								
		Option 1: Antibiotics should be prescribed for tuberculosis * n (%)			Option 2: Antibiotics should be prescribed for tuberculosis and viral illnesses n (%)			Option 3: Antibiotics should be prescribed for viral illnesses n (%)		
		*Pre-test*	*Post-test*	*p value*	*Pre-test*	*Post-test*	*p* value	*Pre-test*	*Post-test*	*p* value
D. Maria II	37	15 (40.5)	27 (73.0)	0.005	22 (59.5)	10 (27.0)	0.005	0 (0.0)	0 (0.0)	-
Camilo Castelo Branco	45	21 (46.7)	36 (80.0)	0.001	24 (53.3)	8 (17.8)	<0.001	0 (0.0)	1 (2.2)	1.000

* Correct answer.

**Table 3:** Percentage of correct answers to questions on antibiotic treatment for bacterial infections.

Study question	Correct answer	D. Maria II (37 students) n (%)			Camilo Castelo Branco (45 students) n (%)		
		*Pre-test*	*Post-test*	*p value*	*Pre-test*	*Post-test*	*p value*
**^1^** Antibiotics do not interact with alcohol	**F**	94.6 (35)	97.3 (36)	0.602	88.9 (40)	95.6 (43)	0.486
**^2^** Antibiotics can be taken at different times each day, if the daily doses are taken	**F**	40.5 (15)	73.0 (27)	0.005	46.7 (21)	82.2 (37)	0.002
**^3^** Antibiotic treatment should be stopped as soon as the patients feels better	**F**	100 (37)	100 (37)	1.000	100 (45)	97.8 (44)	1.000
**^4^** Antibiotics should be shared with other people if the symptoms were similar	**F**	97.3 (36)	97.3 (36)	1.000	95.6 (43)	97.8 (36)	1.000
**^5^** The incorrect use of antibiotics can lead to development of resistant bacteria	**T**	45.9 (17)	62.2 (23)	0.162	51.1 (23)	84.4 (38)	0.162

T = true; F = false.

Comparative of the pre- and post-test for all students from both schools revealed a significant difference for Question 5, with a higher number of correct answers for School Camilo Castelo Branco in the post-test (*p* = 0.028) (Table 4).

**Table 4:** Number of correct answers to questions on antibiotic treatment for bacterial infections.

Study question	Correct answer	D. Maria II/Camilo Castelo Branco (37/45 students) n (%)			D. Maria II/Camilo Castelo Branco (37/45) n (%)		
		*Pre-test*	*Post-test*	*p value*	*Pre-test*	*Post-test*	*p value*
**^1^** Antibiotics do not interact with alcohol	**F**	35 (94.6)	40 (88.9)	0.639	36 (97.3)	43 (95.6)	0.654
**^2^** Antibiotics can be taken at different times each day, if the daily doses are taken	**F**	15 (40.5)	21 (46.7)	0.326	27 (73.0)	37 (82.2)	0.311
**^3^** Antibiotic treatment should be stopped as soon as the patients feels better	**F**	37 (100)	45 (100)	-	37 (100)	44 (97.8)	1.000
**^4^** Antibiotics should be shared with other people if the symptoms were similar	**F**	36 (97.3)	43 (95.6)	1.000	36 (97.3)	44 (97.8)	0.361
**^5^** The incorrect use of antibiotics can lead to development of resistant bacteria	**T**	17 (45.9)	23 (51.1)	0.516	23 (62.2)	38 (84.4)	0.028

T = true; F = false.

## 4. Discussion

This study assessed knowledge of antibiotics among Portuguese school students of the ninth grade and assessed the impact of a teaching intervention on this knowledge. A convenience sample of middle-school students was used to allow for rapid collection of data in a short period of time with limited resources. In this study, a teaching activity on microbiology and infection control was performed, aiming to improve students’ knowledge and attitudes towards antibiotic use.

Educators agree that students do not perform as expected, probably due to the teaching methods used [31]. Although a passive lecture does not promote long-term retention [32], it is still a common approach used by many teachers. However, a previous study showed that by verbalizing their understanding of a topic, a student is “forced” to make its explanation more concrete and specific, thereby promoting long-term memory [33].

The teaching method selected here, a slide show presentation followed by discussion, was carried out after the application of a questionnaire (pre-test). We could see that the students had misunderstandings and lack of knowledge on antibiotics, mainly related to antibiotic spectra and indications. We could see that a considerable number of students believed that antibiotics are effective against virus, fungi, insects, worms and other organisms. Similarly, a study performed in the USA with 5th, 8th and 11th grade students revealed that a common misconception among students was the belief that “antibiotics can cure viral infections” [34].

Concerning the use of antibiotics against bacteria and other organisms, in the current study an increase in the number of correct answers was seen after the teaching intervention (post-test). Another study performed in the USA with undergraduate students involving instruction on microbiology, showed important changes in students’ knowledge on microorganisms and alterations in reported behaviors related to microbial transmission [35]. Similarly, a recent study in Portugal entitled “Microbiology recipes” showed the importance of educational activities incorporating hands-on activities in science education. This project improved the participants’ understanding of bacteria, antibiotics and antibiotic resistance [36].

In the pre-test, students’ knowledge of the value of antibiotics in the treatment of common diseases with different etiologies was limited with the majority responding that antibiotics could be used against viral illnesses (influenza, hepatitis, AIDS), as well as tuberculosis. This result is in accordance with recent studies, which demonstrate that many patients would take antibiotics for a cough and cold symptoms [4,11,37]. The use of antibiotics for treatment of viral illness may be a consequence of bacteria and viruses being misperceived by the general population as identical microorganisms [18,38,39]. Our intervention on this topic was valuable since the students in the post-test revealed a significant increase in the number of correct answers.

With respect to correct antibiotic use, we noticed some gaps in the pre-test, however, the results in the post-test for both schools improved significantly students’ knowledge, (a) in respect to the obligation to comply with the schedule set, with 73% and 82.2% of correct answers respectively and, (b) regarding the development of resistant bacteria with 62.2% and 84.4% of correct answers. These results were very promising since the overuse and misuse of antibiotics may lead to important health problems, primarily antibiotic resistance [5]. After 1994, the resistance shown by *Streptococcus pneumonia* in 13 European countries was correlated with the use of antibiotics. When bacteria become resistant, the time of treatment is prolonged and contributes to the occurrence of side effects and increased treatment costs [40,41]. The lowest levels of resistance have been recorded in Scandinavian countries, Great Britain and Netherlands. This may be due to strict guidelines for antibiotic prescription and use [40,42]. Concerning the need to complete the full course of antibiotics when symptoms of infection are improving, our results were very positive, in agreement with the studies by the groups of Oh and You and collaborators [5,11]. As well as encouraging the use of antibiotics only when necessary, the importance of following the physician’s advice must be stressed, especially in finishing a full course of treatment [42,43].

A limitation of this study is the use of a convenience sample of ninth graders in two schools. While this facilitated the conduct of the study it may limit the generalizability of the findings. Further study in other areas of the country with larger urban and rural populations will help to confirm these findings.

Our study included students from two different schools, one located outside the city of Vila Nova de Famalicão (D. Maria II), and another located in the centre of the same city (Camilo Castelo Branco). One school receives students an urban setting and the second from rural and manufacturing areas. The students from School Camilo Castelo Branco had better results in the pre-test, which may reflect the cultural level of these students compared to students from the School D. Maria II.

A study conducted by Parimi and collaborators [6] revealed that antibiotic knowledge influenced attitudes and behavior towards antibiotic use. Another study demonstrated that demand for antibiotic prescriptions and keeping these antimicrobials at home to treat illnesses was higher in persons with inadequate knowledge [44].

Educational campaigns have had a positive effect on knowledge and reduction in antibiotic prescription in different countries [18,21,45]. Lecky* et al.* have provided a detailed description of the education materials used in the E-Bug project [46]. From the same group, Farrell demonstrated how computer games are effective in increasing knowledge regarding the same learning objectives of infectious diseases and antibiotic use in children [47]. Madle* et al.* have shown that web based teaching materials can change knowledge and attitudes regarding antibiotic use [48]. An educational program directed at both health professionals and the general public in Canada was associated with a decline in antibiotic prescribing rates, as reported by McKay [49].

## 5. Conclusions

Our findings support the idea that correct dissemination of information on antibiotics can change attitudes and behaviors regarding antibiotic use. Regular activities on appropriate antibiotic use are crucial to correct misconceptions on antibiotic use. It is essential to promote student involvement, namely active learning, into traditional teaching, stimulating students intellectually and promoting better learning. Another advantage of school interventions is that they provide the opportunity to reach a large proportion of students in their normal learning environment.

In conclusion, the strategy used in this study seems promising. We believe it can be replicated in other schools, complemented by experimental activities, to promote long-term retention of knowledge. This has been the experience of the E-Bug program, which has also been implemented in Portugal [50]. It is crucial to include a microbiology unit in the curricula of middle-school students to help students to understand the importance of microorganisms in their health. For some students, this will be their last contact with this field of study, since they will pursue other areas of knowledge, such as the arts, the humanities, economics and engineering.
